# Development and Validation of a Nursing Work Interruption Scale

**DOI:** 10.3390/ijerph192013487

**Published:** 2022-10-18

**Authors:** Eun-Jeong Yu, Eun-Nam Lee

**Affiliations:** 1 Nursing Department, Busan Institute of Science and Technology, Busan 6639, Korea; 2 College of Nursing, Dong-A University, Busan 49201, Korea

**Keywords:** nurses, work, factor analysis

## Abstract

Work interruption disturbs nurses’ flow of thinking, diminishes work efficiency, induces burnout, and causes errors that can threaten patients’ lives. Therefore, it is important to identify the causes and measure the extent of work interruption. This study developed a self-report scale and established its validity and reliability for use in hospital settings. Through literature review and in-depth interviews with nurses, we identified two components and developed 25 preliminary items. These items were reviewed by nursing experts for content validity and pilot tested among 20 hospital nurses; subsequently, a 16-item preliminary instrument was finalized. A total of 359 questionnaires were included in the final analysis, and exploratory factor analysis (EFA) and confirmatory factor analysis (CFA) were performed. Two factors and 12 items were derived from two rounds of EFA, with a cumulative percentage of variance of 55.73%. Construct validity was established through CFA. The predictive validity and internal consistency reliability of the developed scale were also established. Thus, the 12-item Work Interruption Measurement Scale for Nurses comprising two domains (human and environmental factors) was developed. This scale can be useful in assessing work interruption experienced by nurses and for developing and assessing the effectiveness of interventions pertaining to nurses’ work interruption.

## 1. Introduction

An interruption is “a break in the performance of a human activity initiated by a source internal or external to the recipient” [1]. It is a complex phenomenon that can cause significant problems for both employees and their organizations and is also pervasive in the nursing environment [2]. It often occurs in relatively quiet and controlled environments such as operating rooms, general wards, and intensive care units, as well as environments where many people work together in open spaces, such as emergency rooms [3,4,5,6].

Interruption of nursing work is unavoidable in hospitals dealing with life-threatening emergencies where emergent situations can occur unpredictably [7]. Hence, nurses may often consider workflow interruptions as a common and trivial matter. However, work interruptions delay work completion time, negatively affect the flow of thoughts, work efficiency [8,9,10,11,12,13], and nurses’ health outcomes including burnout and psychosomatic complaints [14,15,16,17], and threaten the safety of patients due to, for example, medication errors [18,19].

In addition, frequent interruption of nursing work affects the nurse’s work performance, and this frequent interruption leads to the nurse’s work stress and burnout [1,20,21,22]. Therefore, it is necessary to measure the work interruption as a cause of stress and burnout, and the burnout can be used as a criterion variable for the predictive validity test of the work interruption scale.

So far, observation has been mainly used to evaluate work interruption [3,4,15,23,24,25,26]. Observation is advantageous as certain situations or actions can be recorded immediately as they occur. Forsyth et al. [27] used the Work Interruption Tool to identify the characteristics of emergency room nurses’ work interruption. This tool allows observers to capture and record the characteristics of work interruption (cause, priority, and place of work interruption) in real time. Triage Interruptions Assessment Tool was also developed as an observational tool to measure triage interruptions in emergency rooms [28]. Healey et al. [29] observed events that distract or interrupt a urology surgery team in an operating room, and the degree of interruption of a surgery team was assessed by weighting using an 8-point ordinal scale. Most observational studies [3,4,15,23,24,25,26,27,28,29,30] used a semi-structured field note or tally sheet as an observation tool to measure the type and degree of interruption.

However, observation is time consuming and expensive, limits the generalizability of the observations, and is prone to observer subjectivity or prejudice [31]. If the observer is not especially familiar with a nurse’s work, it may affect the quality of collected data, and it may be difficult to understand the clinical perspective of interruption of nursing work, making it difficult to use observation tools. In this respect, it is necessary to develop valid and reliable self-reporting measures to assess the degree of workplace interruption.

So far, there have been several attempts to assess workplace interruptions using self-reported measures. The first is a single item in the Organizational Constraints Scale developed by Spector and Jex that specifically assesses the frequency with which “you find it difficult or impossible to do your job because of interruptions by other people” [32]. The second is a four-item scale assessing intrusions [33]. The third is the Workplace Interruption Measure (WIM) developed by Wilkes et al. [34]. It is a more comprehensive measure of all interruption dimensions based on Jett and George’s [35] four-factor conceptual typology of interruptions: intrusions, distractions, discrepancy detections, and breaks. Indeed, Jett and George [35] suggested that each type of interruption can have both positive and negative consequences. Therefore, they recommended developing a distinct scale for each type of interruption. Moreover, since WIM was developed in the general workplace, items such as “when I was tired of work, I took a break” and “I took a rest when I needed it” do not reflect the hospital environment with a structurally different work environment.

In other words, WIM is not suitable for the purpose of this study, which is to measure the degree of nursing work interruption, under the premise that nursing work interruption can lead to negative consequences.

To our knowledge, this study is the first to develop a self-report scale for measuring the degree of nursing work interruption with a focus on the cause of the interruption under the premise that nursing work interruption leads to negative consequences and to evaluate the validity and reliability of the developed scale.

## 2. Materials and Methods

### 2.1. Scale Development

#### 2.1.1. Identifying the Constructs of Work Interruption

We conducted a literature review and in-depth interviews with nurses to identify the constructs related to the causes of interruptions of nursing work.

##### Literature Review

In order to confirm the conceptual definition of nursing work interruption with negative consequences and the construct factors of the scale, a systematic review was conducted. For literature search, National Assembly Library, Korea Education and Research Information Service, Korean Academic Information, MEDLINE, EMBASE, and CINAHL were used. The keywords used in the data search included combinations of “interrupt*” OR “multi-tasking” OR “nurs*”, “interrupt” OR “stop work” OR “nurse”. A total of 886 studies were searched. Through a review of their titles and abstracts, studies that were not related to nursing work such as drug discontinuation and disease treatment discontinuation and duplicate studies were deleted, resulting in 32 articles related to nursing work interruption being selected.

Through a systematic review of previous studies, the concept of interruption of nursing work was defined as “to stop working due to the occurrence of an unexpected internal or external event”. It was confirmed that the interruption of work experienced by nurses was caused by situations such as someone intruding to make a direct request or a phone call from someone [2,12,15,16,18,25,36] and by environmental factors—conversations with others, background noises at the workplace, or beeping sounds made by the equipment—causing distractions [8,12,19,33,34,37].

##### Interviews with Nurses

One-on-one, in-depth interviews were conducted until the data were saturated. Respondents included 13 hospital nurses from different departments, selected to account for varying experiences. There were eight nurses from the emergency room, one from the intensive care unit, three from the internal medicine ward, and one from the outpatient clinic, each with a work experience of 1–17 years. All participants were women aged 24–40 years. Before the interview, participants were briefly introduced to the concept of nursing work interruption and given time to organize their thoughts. Interview questions included, “Tell us about your experiences with interruptions in your nursing work,” “What do you think interrupted your nursing workflow?”, and “In what cases do you think your nursing work was interrupted?”.

The interview data of 13 participants were analyzed using content analysis, which involved two nursing professors with extensive experience in qualitative research discussing the data until reaching a consensus. Consequently, 44 meaningful statements related to the interruption of nursing work were identified, categories were created through a coding process that described words or name titles, and, finally, two themes were derived: human and environmental factors. One category (intentional intervention) and eight subcategories were derived for “human factors,” and three categories (noise that interferes with concentration, unbearable change, and priority change) and nine subcategories were derived for “environmental factors”.

Human factors identified as interruptions in nursing workflow were: questions or requests from patients, caregivers, other nurses, doctors, other department staff, and visitors; phone calls; and instructions from senior nurses.

Further, environmental factors identified as interruptions in nursing workflow included noises such as the noise of machinery and people’s conversations and the low but consistent sound of medical equipment that interfered with the nurses’ concentration. Other interruptions in work were due to overwhelming changes such as several things occurring simultaneously, a sudden deterioration in a patient’s condition, and an increase in the number of tasks to be attended to because of insufficient workforce. Additionally, participants reported that they had to stop working and prioritize tasks such as a safety accident or an emergency patient.

In summary, nursing work interruption was defined as “a hindrance in a nursing task in progress due to human or environmental factors” in this study, and the constructs for the scale for nursing work interruption were identified as “human factors” and “environmental factors”.

#### 2.1.2. Testing the Content Validity

In order to evaluate the content validity of the initial 25 questions, a professional panel consisting of nursing professors with more than 3 years of clinical experience was used. The number of members was 5 according to the criteria suggested by Lynn [38] from 5 or more to 10 people. The content validity was verified by calculating the content validity index (CVI). The CVI was calculated by evaluating how well each question measured nursing workflow interruption on a 4-point Likert scale. An item was revised if it was inappropriate, or a free opinion was written. According to Lynn [38], the CVI should be 1.0 when an expert group comprises three members at the time of the content validity test. Hence, nine questions with a CVI of 1.0 or less were deleted and redundant or ambiguous items were corrected; 16 pilot questions were finally formed.

#### 2.1.3. Pilot Survey

The subjects of the pilot survey were nurses with more than six months of experience in performing independent nursing in their working hospitals, according to the criteria of DeVellis [39] and 20 to 40 subjects with similar characteristics to those in the present study were deemed suitable. In order to collect various opinions, there was no restriction on the working department, and it was conducted on 20 nurses at Dong-A University Hospital who understood the purpose of the pilot survey.

All participants were women, with an average age of 31 ± 7.9 years; furthermore, 80% were single, and 80% had a bachelor’s degree. They had an average hospital work experience of 7.9 ± 7.7 years and worked in the intensive care unit (40%), emergency room (25%), internal medicine ward (25%), or other departments. Participants included 19 general nurses and one charge nurse. In the pilot survey, it was suggested that the frequency of work interruption needed to be increased. The average frequency of interruption of nursing work in the pilot test was mainly distributed from “more than 10 times a week” to “once to three times a week”.

#### 2.1.4. Scale Response Format

We decided the scale response options based on previous studies [3,5,25,32,34,37] and pilot test results. The Organization Constraints Scale consists of categories “once a month or no,” “1–2 times a month,” “1–2 times a week,” “1–2 times a day,” and “several times a day”. The WIM consists of categories “none for the past week,” “1–3 times a week,” “4–6 times a week,” “7–9 times a week,” and “more than 10 times a week”. Previous studies observing the frequency of nursing interruption reported 3–7 occurrences per hour based on an 8-h work per day, but these studies were considered to be different from this study as a result of observing emergency rooms and intensive care units. In the pilot test, the average frequency of interruption of nursing work mainly ranged from “more than 10 times a week” to “once to three times a week”.

Based on the pilot survey, we revised the scale’s frequency response options along a 6-point scale, comprising the options “almost never,” “once or twice a week,” “three to four times a week,” “once or twice a day,” “three to four times a day,” and “more than five times a day”.

### 2.2. Validation of the Scale

#### 2.2.1. Setting and Sample

To verify the validity and reliability of the scale, the main survey was conducted among nurses working in tertiary care or general hospitals in Busan. Study participants were nurses who have at least six months’ experience in performing independent nursing, and there was no restriction on the working period or position. It was expected that the type of nursing work would differ depending on the working department or the working period, so we confirmed the interruption of nursing work with various participants. However, participants working in the central supply rooms, outpatients, operating rooms, and insurance screening departments that could be replaced by personnel other than professional nurses were excluded. 

The sample size of this study was based on the criteria that more than five times the number of questions [40] was appropriate. Factor analysis is required for scale development, reflecting the opinion that 150 to 200 people are suitable for exploratory factor analysis [41] and at least 150 samples are suitable for confirmatory factor analysis [42]. Therefore, to satisfy all requirements, a total of 370 subjects were recruited by considering the dropout rate of 15% after applying 10 multiples of 16 questions, and to organize the subjects of exploratory factor analysis and confirmatory factor analysis differently.

Among the 370 distributed questionnaires, 369 were collected; 359 copies were used for the final analyses, excluding 10 copies with unanswered items.

Data were collected between 12 August and 8 September 2019. The purpose of the study was explained to the nursing administration department of hospitals, and cooperation was sought to include participants with varying work experiences from different departments. The questionnaires were distributed to and filled out by nurses who had provided consent to participate in the study after reading the recruitment announcement.

#### 2.2.2. Evaluating the Predictive Validity

The scale’s correlation with work demand was measured to verify the extent to which the work interruption scale predicted future outcomes. Frequent nursing work interruptions increase work demand among nurses [12,13,37,40,43]. Therefore, the Korean version of the National Aeronautics and Space Administration Task Load Index (NASA-TLX) (paper and pencil version) [44], which is a numeric rating scale developed to measure the work demand of workers, was used in this study to evaluate predictive validity.

NASA-TLX comprises six items: mental demand, physical demand, temporal demand, frustration, effort, and performance. The scale of each item consisted of 20 equal intervals. The score on each scale showed the level of work demand that nurses experienced, and the total work demand was the sum of the scores of each item. The scores ranged from 0 to 100 points, and the higher the score, the greater the burden of work. In Park and Lee’s study [44], Cronbach’s alpha was 0.88, whereas, in this study, it was 0.87.

### 2.3. Data Analyses

The collected data were analyzed using IBM SPSS/WIN 23.0 and AMOS/WIN 24.0 (IBM Inc., Armonk, NY, USA). The general characteristics of the participants were analyzed using descriptive statistics. The mean and standard deviation of the items were calculated for item analysis; skewness and kurtosis were obtained to evaluate normality; and the revised item-total correlation coefficient was calculated. EFA and CFA were conducted to test the validity of the composition. In EFA, we performed a principal component analysis suitable to facilitate interpretation by reducing the dimension of the measured variables. For factor rotation, varimax rotation was used [45]. The number of factors was determined based on the scree plot and the cumulative explained variance ratio. In CFA, χ^2^, χ^2^/df, goodness-of-fit index (GFI), root mean square error of approximation (RMSEA), normed fit index (NFI), comparative fit index (CFI), and Tucker–Lewis Index (TLI) were calculated to evaluate the goodness-of-fit of the model. Pearson correlation coefficient with the Korean version of NASA-TLX score was used to determine predictive validity. Cronbach’s α coefficient was calculated to test reliability.

### 2.4. Ethical Considerations

This study was approved by the Institutional Review Board of Dong-A University (1040709-AB-N-01-201906-BR-004-04). The measurement tool was used after obtaining permission from the original tool developer and the domestic author who modified it via email. Regarding the survey, the nurses were informed about the purpose and method of the study, and those who voluntarily agreed to participate were included.

## 3. Results

### 3.1. Characteristics of the Participants

The mean age of the participants was 29 years; furthermore, 94.4% were women, 78.8% were unmarried, 81.3% had a bachelor’s degree, and 58.5% were not religious. The participants had a mean nursing experience of 6.4 years—59.4% had less than five years of experience and 21.5% had 10 or more years of experience. The majority of the participants worked in the medical/surgical wards (57.1%), followed by the intensive care unit (29.2%) and emergency room (13.6%). Most of the nurses were staff nurses (89.4%), and 10.6% were charge and head nurses.

The 359 participants were divided into two groups using random sampling through a computer; EFA was conducted with the data of one group (200 participants), and CFA was conducted with the data of the other group (159 participants; Table 1).

### 3.2. Item Analysis

The item analysis of 16 items showed that the mean score ranged from 2.95 to 5.41, skewness ranged from −1.97 to 0.75, and kurtosis ranged from −1.16 to 4.37. There were no items outside the standard values of ±3 skewness and ±7 kurtosis, and the items could be assumed to have a normal distribution [46]. All corrected item-total correlation coefficients had values of 0.30 or higher [47], ranging from 0.38 to 0.69.

### 3.3. Validity of the Scale

#### 3.3.1. Construct Validity

Construct validity of the scale was determined using EFA and CFA.

##### Exploratory Factor Analysis

The Kaiser–Meyer–Olkin (KMO) test and Bartlett’s sphericity test were performed to examine whether the collected data were adequate for EFA. The KMO value was high at 0.87, and Bartlett’s sphericity test result showed that χ^2^ = 1339.67, *p* < 0.001, indicating that the data were adequate for performing EFA. 

In the scree plot, the number of factors was set to two as the graph became gentle at the point where the factor was 3, with a cumulative explained variance of 49.5%.

In the EFA, the maximum loading was 0.40 or more for all items; however, four items were deleted as items 4, 7, and 16 scored under 0.40 for Communality, and item 9 had redundant loadings on two or more factors.

After deleting four items in the primary EFA, a secondary EFA was performed for the remaining 12 items, which were consequently classified into two factors, with a cumulative explained variance of 55.7%. The factor analysis found that the maximum loading was 0.50 or more for all items, and the Communality was 0.40 or more.

Among the 12 items finally selected after the EFA, factor 1 consisted of six items, an eigenvalue of 3.26, and explained variance of 27.1%; and factor 2 consisted of six items, an eigenvalue of 3.43, and explained variance of 28.6% (Table 2).

Based on the literature review [3,5,11,15,17,23,24,25,26,37,43,48], the typology of work interruption of Jett and George [35], and the concept analysis of Brixey et al. [1], it was found that nursing work interruption was mainly caused by people such as fellow nurses, doctors, patients, and caregivers or the working environment. So, factor 1 was named “human factors” as it comprised items related to the unexpected intentional intrusion by another person; factor 2 was named “environmental factors” as it comprised items related to the surroundings that affected concentration during work.

##### Confirmatory Factor Analysis

The CFA was performed on the two factors and 12 items of the work interruption scale. On verifying the goodness-of-fit of the model, GFI was 0.88, TLI was 0.89, CFI was 0.91, NFI was 0.86, and RMSEA was 0.09, thus satisfying the criteria for goodness-of-fit. The standardized regression coefficient for convergent validity was 0.50 or higher for all items of factors 1 (human factors) and 2 (environmental factors). The average variance extracted (AVE) of all factors was also high (above 0.50), and the construct reliability (CR) was above 0.70. To determine the discriminant validity, the AVE and the square of the correlation coefficient were compared. The AVE ranged from 0.50 to 0.51, whereas the square of the largest correlation coefficient was 0.44, with all the AVE values greater than the square of the correlation coefficients, indicating that the concepts between factors were separate from each other (Table 3).

#### 3.3.2. Predictive Validity

We examined the scores of the Korean version of the NASA-TLX and Pearson’s correlation coefficient to verify the predictive validity, which was confirmed through a positive correlation of 0.42 (*p* < 0.001).

### 3.4. Reliability of the Scale

On examining internal consistency, we found Cronbach’s ⍺ values of 0.88 for all 12 items, 0.84 for factor 1 (human factors), and 0.83 for factor 2 (environmental factors), all demonstrating high reliability.

Thus, the Nursing Work Interruption Scale was developed once its validity and reliability had been verified (Table 4).

## 4. Discussion

This study is meaningful in that it is the first, to the best of our knowledge, to develop a self-report measurement tool of the interruption of nursing work that reflects the specificity of the nursing work environment. The nursing work interruption measurement tool developed in this study comprised 12 items based on two factors identified, and its total explanatory power was 55.7%, which is slightly lower than that of the WIM [34]. This is thought to be due to the difference in the number of factors. However, it is difficult to compare directly because the WIM consists of four factors, including two factors of work interruption (discrepancy detection and break) that lead to positive consequences. Meanwhile, Lin et al. [33] who measured the degree of work interruption with only four items representing intrusion, showed 69.9% of variance. There are differences in explanatory power, but intrusion can be considered as the main type of work interruption not only in general workplaces but also in the nursing environment.

During EFA, the item, “I had to stop what I was doing to handle a task given by a senior nurse,” was deleted because of its similarity to the item, “I had to stop what I was doing as my fellow nurse asked for something”. Further, the item, “I had to stop what I was doing to answer the phone,” was also deleted due to its poor explanatory power. In nursing work, the phone is the main medium for interacting with individuals from different departments and for exchanging information. Therefore, this item was deleted as it included both human factors related to the callers who had certain requests and environmental factors such as frequent ringtones and individuals talking on the phone. Additionally, the item, “I had to stop what I was doing to check an incorrect prescription by a doctor,” did not accurately describe intrusion by someone with a certain intention. In contrast, the presentation of a prescription by a doctor could be interpreted as a request from an individual. Therefore, it is necessary to distinguish these characteristics more clearly through further studies.

Nursing work interruption showed a significant positive correlation with NASA-TLX [44], thus, confirming that the more the work interruption, the higher the work burden. Lin et al. [33] suggested that intrusion can be disruptive to an employee because it displaces the time that employee needs to complete a task, which increases their perceived workload. It was also said that this workload leads to work strain. In addition, Spector and Jex [32] and Wilkes et al. [34] also pointed out that frequent interruption leads to an increase in workload, but conversely, an increase in workload can also increase interruption.

The factors of the nursing work interruption measurement tool developed through this study partially matched the factors of the WIM that measure the interruption of work in a general office. Wilkes et al. [34] developed the WIM based on the four types of interruptions proposed by Jett and George [35]. On the other hand, since this study developed a tool focusing on the causes of nursing interruption with negative consequences, it did not include the type of break that leads to positive consequences and discrepancy detection that prevents negative consequences by noticing something wrong. In addition, unlike general workplaces, hospitals where nurses work do not have an environment where they can take a break to recover their condition while performing nursing work, and stopping work because of mistakes has positive consequences such as preventing medical accidents. In addition, to reflect the characteristics of nursing work and the environment of hospitals, items, “I stopped working due to sudden changes in a patient’s condition,” “I stopped working due to a sudden increase in the number of patients in my charge,” and “I stopped working due to the alarm sounds of a medical equipment or machines” were included. In this respect, we think this measurement tool is suitable for measuring nursing interruption that reflects the specificity of nursing work without deviating from the nature of general workplace interruption itself.

The “human factor,” the first component of the nursing work disruption measurement tool developed through this study, refers to a person who intends to stop someone’s work through direct intervention and who affects the flow of work by appearing unexpectedly and disrupting work [35]. “Intrusions” is a type of interruption proposed by Jett and George [35], which shortens the time available to a person to perform their original work due to unplanned interactions between individuals, resulting in the person taking a longer time to complete their work. 

Brixey et al. [1] introduced “intrusion” as a major attribute of workflow interruption and defined it as an invasion by another person into the workspace at any time while performing a task. Work interruption due to intrusion has been introduced as a common phenomenon in the modern work environment [33]. In a concept analysis [49], “intrusion” was identified as the main attribute of work interruption. Wilkes et al. [34] also implicated “intrusion” majorly in workflow interruption, and items such as, “I had to stop working on my task because someone suddenly gave me a new job” and “I had to stop working on my task at the request of a colleague at work” were related to the human factor. In this study, people who cause interruptions were specified as patients, doctors, fellow nurses, other employees, and guardians to reflect the specificity of the hospital environment.

Among the human factors of the developed scale, the prevalence of the item “I had to stop what I was doing to answer the phone” was the most frequent, with an average of more than five times a day, followed by the item “I had to stop what I was doing because the patient suddenly asked for something”. The “human factor” is not recognized as a workflow interruption in a place where the nature of the work is to take care of someone. However, it is necessary to inform patients and caregivers visiting the hospital about the negative impact of nurse interruption on their work. In addition, it is necessary to improve the misconception that it is natural for nurses to stop their work or that it is always permissible to interrupt nurses. Along with this improvement in awareness, it is suggested that hospital policy dimensions such as designating areas in which important tasks are performed as ‘no-interruption zones’ or employing dedicated personnel to address calls or demands. The “human factor” is often not recognized as a workflow interruption since the nature of nursing work is to take care of someone. The results of this study suggest that apart from nurses, other medical staff should also recognize the occurrence and negative effects of nursing work interruptions and determine measures to reduce interruptions.

The second factor or the “environmental factor” refers to a phenomenon in which a stimulus resulting from changes in an environment distracts or interferes with a nurse’s concentration. Distractions are cognitive changes caused by external stimuli or secondary tasks that cause personal psychological reactions that disrupt the workflow [35]. Distractions are representative of work interruptions resulting from the environment [11,15,34,50]. Among the scale developed in this study, the prevalence of environmental factors was most frequently “stopped work due to multiple people’s requests” at an average of 3–4 times a day, followed by “stopped work with the alarm sound of medical equipment or machines” at an average of 1–2 times a day. Particularly, noise is a distractor in most work environments, and in hospital environments, it is caused by an ongoing communication among doctors, nurses, patients, and families of patients as well as the operation of medical equipment [51]. At times, the sound can be extremely frequent or loud, which can be annoying and distracting for the individuals working in that environment. 

In Wilkes et al.’s study [34], only “noises” were included in the items about environmental distractions such as, “I was distracted by the background noise while working” and “I had to adjust the distracting noise while working”. In this study, the items on distractions also included background noises and the uncontrollable hospital work environment such as “a sudden change in a patient’s condition,” “alarm sound from a medical equipment or machinery,” “equipment malfunction,” and “a sudden increase in the number of patients”. 

Through a literature review and qualitative interviews, statements such as “Because peer nurses or doctors make noise…”, “Because caregivers make noise…”, and “Because more patients have to use medical devices…” were derived, but they were deleted due to overlapping expressions with other questions as a result from the content validity test. However, in subsequent studies, it is expected that the questions will be more clarified and applied with objectivity.

The scale developed in this study can be useful for identifying the cause of the work interruptions for nurses working at a hospital regardless of their working departments. Specifically, this scale could be useful for association analysis and verification of the effectiveness of countermeasures in departments with frequent resignations, where nurses are reluctant to work, and with frequent work-related errors. It can also be used to collect basic data to investigate work demand, work stress, and burnout among nurses, and to analyze nursing work.

This study has several limitations. First, the participants were recruited from three hospitals using convenience sampling. Therefore, caution is required while generalizing the results to nurses in all hospitals; moreover, future studies are required to corroborate our results. Second, since the questionnaire was filled in by recalling memories of the events during the past week, the results may not reflect the actual frequency of their occurrence. During the current circumstances, as there is a shortage of nursing manpower in Korea, objectively analyzing the nursing work interruption could contribute toward the effective management of human resources. The tool developed in this study could help nurses by establishing a better working environment; reducing work demand, stress, and burnout; and increasing job satisfaction. This study could not reflect changes in the hospital environment because data were collected before the occurrence of the COVID-19 pandemic. It is hoped that the developed scale in the follow-up study will be used to identify the difference in the degree of interruption after the occurrence of the pandemic and that new items will be added related to visit restrictions, noise in the work environment, changes in disease severity, and changes in the nursing system for infection control.

## 5. Conclusions

The final developed scale for interruption of nursing work consisted of 12 questions, two components of “human factors” and “environmental factors”, and the higher the score, the more frequent the perceived interruption of nursing work. The developed scale in this study is a tool with verified validity and reliability and it will be useful for specifically and accurately evaluating the actual phenomenon of interruption in nursing work. In addition, by objectively measuring the environment in which hospital nurses work, it will be possible to contribute to the management of nursing personnel, patient safety, and improvement of nursing organization’s productivity. 

## Figures and Tables

**Table 1:** Demographic characteristics of participants.

Characteristics	Categories	Total (N = 359) n (%)	EFA (N = 200) n (%)	CFA (N = 159) n (%)
Gender	Men	20 (5.6)	11 (5.5)	9 (5.7)
	Women	339 (94.4)	189 (94.5)	150 (94.3)
Age (year)	<25	68 (18.9)	40 (20.0)	28 (17.6)
	25–29	187 (52.1)	96 (48.0)	91 (57.2)
	30–34	33 (9.2)	21 (10.5)	12 (7.5)
	35–39	34 (9.5)	19 (9.5)	15 (9.4)
	40≤	37 (10.3)	24 (12.0)	13 (8.2)
	Mean ± SD (Range)	29.17 ± 6.49 (22.54)	29.48 ± 6.63 (22.54)	28.78 ± 6.31 (22.54)
Marital status	Married	76 (21.2)	44 (22.0)	32 (20.1)
	Unmarried	283 (78.8)	156 (78.0)	127 (79.9)
Religion	No	210 (58.5)	109 (54.5)	101 (63.5)
	Yes	149 (41.5)	91 (45.5)	58 (36.5)
Education	College	41 (11.4)	21 (10.5)	20 (12.6)
	University (RN-BSN)	292 (81.3)	161 (80.5)	131 (82.4)
	Graduate school	26 (7.2)	18 (9.0)	8 (5.0)
Nursing experience (year)	<2	85 (23.7)	50 (25.0)	35 (22.0)
	2–4	128 (35.7)	63 (31.5)	65 (40.9)
	5–9	69 (19.2)	40 (20.0)	29 (18.2)
	10–14	29 (8.1)	17 (8.5)	12 (7.5)
	15 ≤	48 (13.4)	30 (15.0)	18 (11.3)
	Mean ± SD (Range)	6.40 ± 6.62 (0.08–33.50)	6.63 ± 6.75 (17–33.50)	6.10 ± 6.47 (0.08–33.50)
Department	Internal medicine ward	130 (36.2)	63 (31.5)	67 (42.1)
	Surgical ward	75 (20.9)	46 (23.0)	29 (18.2)
	Emergency room	49 (13.6)	24 (12.0)	25 (15.7)
	Intensive care unit	106 (29.2)	67 (33.5)	38 (23.9)
Type of duty	Shift	336 (93.6)	184 (92.0)	152 (95.6)
	Non-shift	23 (6.4)	16 (8.0)	7 (4.4)
Position	Staff	321 (89.4)	176 (88.0)	145 (91.2)
	Charge or head nurse	38 (10.6)	24 (12.0)	14 (8.8)
Workplace	University hospital	302 (84.1)	166 (83.0)	136 (85.5)
	General hospital	57 (15.9)	34 (17.0)	23 (14.5)
	Total	359 (100.0)	200 (100.0)	159 (100.0)

EFA, Exploratory factor analysis; CFA, Confirmatory factor analysis.

**Table 2:** Exploratory factor analysis (N = 200).

Item No.	Contents	Factors		Communality
		1	2	
06	I had to stop what I was doing due to a guardian suddenly asking for something.	**0.79**	0.02	0.62
01	I had to stop what I was doing due to a patient suddenly asking for something.	**0.79**	−0.06	0.62
05	I had to stop what I was doing due to an employee from another department suddenly asking for something.	**0.70**	0.33	0.59
03	I had to stop what I was doing due to a colleague (nurse) suddenly asking for something.	**0.68**	0.25	0.53
02	I had to stop what I was doing due to a doctor suddenly asking for something.	**0.62**	0.27	0.46
13	I had to stop what I was doing due to several people asking for something at the same time.	**0.57**	0.48	0.56
11	I had to stop what I was doing due to a sudden change in a patient’s condition.	0.28	**0.76**	0.65
14	I had to stop what I was doing due to the alarm sound of a medical equipment or machinery.	0.05	**0.76**	0.57
15	I had to stop what I was doing due to a malfunction of an equipment.	0.02	**0.75**	0.56
08	I had to stop what I was doing due to an emergency (safety accident, fire, theft, etc.).	0.09	**0.66**	0.44
12	I had to stop what I was doing due to a sudden increase in the number of patients.	0.38	**0.64**	0.56
10	I had to stop what I was doing due to the sudden fuss of a patient and/or their guardian.	0.34	**0.63**	0.52
Eigen value		3.26	3.43	
Explained variance (%)		27.1	28.6	
Total explained variance (%)		55.7		

**Table 3:** Confirmatory factor analysis (N = 159).

Factors			Item No.	SRW	CR	AVE	Construct Reliability		
Human factors			01	0.71		0.51	0.86		
			02	0.7	8.08				
			03	0.68	7.82				
			05	0.77	8.80				
			06	0.7	8.09				
			13	0.71	8.17				
Environmental factors			08	0.58		0.50	0.86		
			10	0.79	7.29				
			11	0.85	7.54				
			12	0.67	6.56				
			14	0.58	5.95				
			15	0.70	6.77				
Fitness index	χ^2^(p)	χ^2^/df	GFI	TLI	RMSEA	NFI	CFI	PNFI	PCFI
Criteria	>0.05	≤3	≥0.80	≥0.80	≤0.10	≥0.80	≥0.80	0.60~0.90	≥0.50
Model	131.08 (<0.001)	2.47	0.88	0.89	0.09	0.86	0.91	0.69	0.73

AVE, Average variance extracted; CFI, Comparative fit index; CR, Critical ratio; GFI, Goodness-of-fit index; NFI, Normed fit index; PCFI, Parsimonious comparative fit index; PNFI, Parsimonious normed fit index; RMSEA, Root mean square error of approximation; SRW, Standard regression weight; TLI, Tucker–Lewis index.

**Table 4:** The Nursing Work Interruption Scale (Final).

Item No.	Contents	At Least 5 Times per Day	Average 3–4 Times per Day	Average 1–2 Time(s) per Day	3–4 Times a Week	1–2 Time(s) a Week	Almost None
1	I had to stop what I was doing due to a patient suddenly asking for something.	6	5	4	3	2	1
2	I had to stop what I was doing due to a doctor suddenly asking for something.	6	5	4	3	2	1
3	I had to stop what I was doing due to a colleague (nurse) suddenly asking for something.	6	5	4	3	2	1
4	I had to stop what I was doing due to an employee from another department suddenly asking for something.	6	5	4	3	2	1
5	I had to stop what I was doing due to a guardian suddenly asking for something.	6	5	4	3	2	1
6	I had to stop what I was doing due to several people asking for something at the same time.	6	5	4	3	2	1
7	I had to stop what I was doing due to an emergency (safety accident, fire, theft, etc.).	6	5	4	3	2	1
8	I had to stop what I was doing due to the sudden fuss of a patient and/or their guardian.	6	5	4	3	2	1
9	I had to stop what I was doing due to a sudden change in a patient’s condition.	6	5	4	3	2	1
10	I had to stop what I was doing due to a sudden increase in the number of patients.	6	5	4	3	2	1
11	I had to stop what I was doing due to the sound of an alarm from a medical device or machine.	6	5	4	3	2	1
12	I had to stop what I was doing due to a malfunction of an equipment.	6	5	4	3	2	1

## Data Availability

All data that support the findings of this study are available from the corresponding author upon reasonable request.
